# Complete pathological response in a patient with multiple liver metastases from colon cancer treated with Folfox-6 chemotherapy plus bevacizumab: a case report

**DOI:** 10.1186/1756-8722-2-35

**Published:** 2009-08-06

**Authors:** Norma Malavasi, Giovanni Ponti, Roberta Depenni, Federica Bertolini, Sandra Zironi, Gabriele Luppi, Pier Franco Conte

**Affiliations:** 1 University of Modena and Reggio Emilia, Department of Oncology and Haematology, via del Pozzo, 71; 41100 Modena, Italy

## Abstract

The complete pathological response after primary chemotherapy could represent an important prognostic factor in patients affected by colorectal liver metastases.

In recent studies, increasing complete pathological response seems to be correlated with longer overall survival periods and it is recognized as an important prognostic factor in patients treated with pre-operative chemotherapy.

The correlation of radiological information on residual neoplastic disease after neoadjuvant treatment, obtained with CT and PET, has to be evaluated; in fact the complete disappearance of liver metastasis on radiological imaging does not always mean a complete disappearance of tumor tissue on histological examination; when it is documented with surgical procedures and confirmed by pathologist's examination, we can consider the complete pathological response.

In recent years the addition of monoclonal antibodies to conventional chemotherapy may further increase the proportion of patients referred for surgery; bevacizumab before surgery has been shown to be feasible and safe, although concerns still exist regarding possible post-surgical and wound healing complications or bleeding. The limitation of the radiologic assessment of response as a surrogate for pathological response is even more relevant when antiangiogenic treatments are used. Excellent responses to bevacizumab-containing regimens do occur and referral to surgical oncology is a crucial step for documentation of complete pathological response.

## Background

At present, the only available treatment associated with long-term survival in patients with colorectal cancer metastases is liver resection with 5-year survival rates ranging from 21% to 58%[1]. Unfortunately, only 10% to 25% of patients with colorectal liver metastases are eligible for surgical resection. The standard of care in unresectable patients is palliative chemotherapy in order to improve overall survival; however, chemotherapy may also be used in an attempt to render liver metastases amenable to surgical resection. Thanks to systemic chemotherapy, resections of initially unresectable liver metastases have been reported in about 13% of patients [2] with successful 5-year overall survival comparable to patients primarily respectable[3].

In resectable patients, pre-operative chemotherapy may increase the R0 resection rate and facilitate limited hepatectomies, hence sparing normal liver parenchyma and improving post-operative recovery[4]. The objective of this approach is also to control the metastatic disease in order to avoid surgery in patients with rapidly progressive disease associated with a poor outcome after hepatic resection[5]. Progressively, pCR seems to be correlated with longer overall survival periods and is recognized as an important prognostic factor in patients treated with pre-operative chemotherapy for breast, esophageal, gastric and rectal cancer primitive tumors [6,7].

Interestingly, the pCR, still reported as a rare situation with an overall incidence of 4% of all resected patients, is going to achieve clinical significance implying the complete absence of residual neoplastic tissue on examination by a pathologist [8]. In a recent study by Adam *et al.*, the pCR of liver metastases was associated with a 5-year overall survival of 76%[9].

Complete metabolic response on PET scan after neoadjuvant chemotherapy is not always a reliable indicator of pCR. Even though the PET scan has the advantage of combining functional and anatomic imaging in an integrated scanner, discordant data from the literature indicate the limitations of the PET scan in restaging patients with hepatic colorectal metastases following neoadjuvant chemotherapy; surgical decision-making often requires information from multiple modalities. Lesions not seen on imaging are still found to have viable tumors when resected or to lead to recurrence without resection[10]. pCR is described as being more frequent than CR, indicating that total necrosis of tumor cells does not imply disappearance of metastasis in pre-operative imaging and does not necessarily correspond to CR[9].

In recent years, novel biological agents have also changed the standard of care for metastatic colorectal cancer and may have implications for neoadjuvant treatment The limitation of the radiologic assessment of response as a surrogate for pathological response is even more relevant when antiangiogenic treatments are used.

We report a case of pCR after primary chemotherapy of four courses of FOLFOX-6 plus bevacizumab (much shorter than expected because of poor tolerability) of colorectal liver metastases confirmed by laparoscopic liver biopsies; CT and PET scans showed good correspondence between the two imaging techniques and between clinical and pathological response. After 36 months, the patient is alive and disease free.

## Case presentation

**Figure 1:**
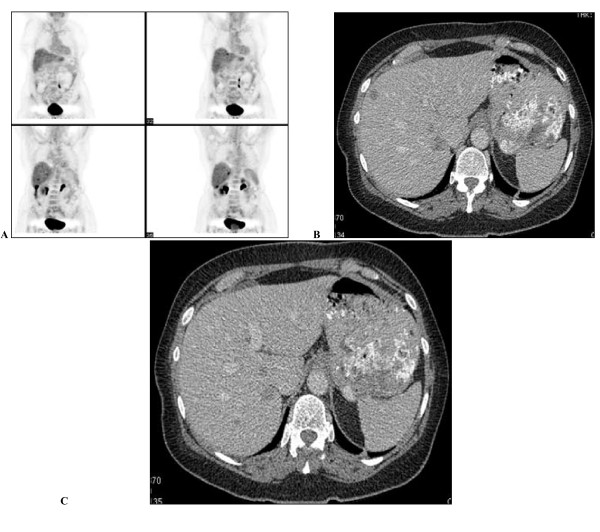
**a, b, c – Imaging before primary treatment: multiple liver metastases and no extra hepatic disease described by CT and PET**.

In June 2006, a healthy 72-year-old woman presented with rectal bleeding, which had started a few weeks before, and without significant anemia or clinical symptoms. In her medical history, no prior pathological conditions, no familial cancer or concomitant medications were reported. Endoscopic examination revealed a voluminous neoplastic mass in the cecal tract of the colon without obstruction; a biopsy of the lesion established the diagnosis of adenocarcinoma. At computed tomography (CT) scan, multiple liver metastases (16 lesions with 1.5 cm as the largest diameter) were detected. The patient underwent a right hemicolectomy and the pathology report showed a moderately differentiated adenocarcinoma of the cecal tract invading the adipose perivisceral tissue with metastatic involvement in one of the 23 nodes removed (pT3N1). Surgical biopsy of a liver lesion confirmed the presence of metastatic disease. A positron emission tomography (PET) scan showed a standardized uptake value (SUV) of ≥8 of liver lesions and the absence of extra-hepatic uptake (Fig. 1). The patient was entered into a phase II clinical trial of FOLFOX-6 plus bevacizumab 5 mg/kg, every 2 weeks. After the first course of therapy, the patient experienced a G3 neutropenia and subsequent chemotherapy doses were reduced to 75% while maintaining the initially planned dose of bevacizumab. In spite of dose reduction, the patient again experienced a G3 neutropenia, G3 diarrhea and abdominal pain requiring hospitalization.

**Figure 2:**
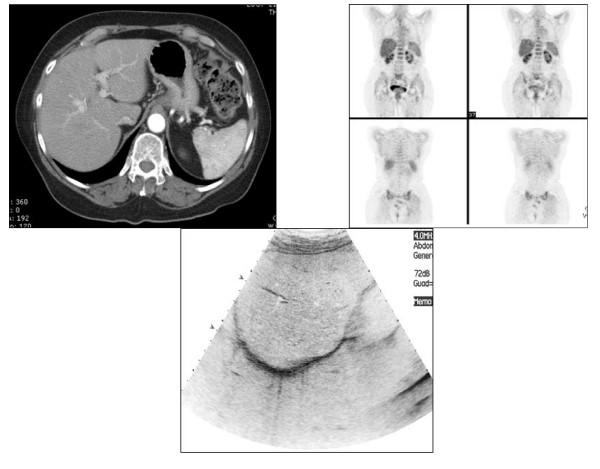
**a, b, c – Imaging after 4 courses of primary treatment, cCR described by CT, PET and liver ultrasound with contrast**.

Because of poor tolerability, a CT scan and a PET scan were performed after four courses of treatment (instead of the initially planned six courses) and did not reveal any liver metastases (Fig 2). The patient was referred to a surgeon: a laparoscopy was performed with ultrasound-guided multiple liver biopsies. At pathology, no tumor cells were detected. Because of the documented pathological CR and prior toxicities, no further therapy was given. After 36 months, the patient is alive and disease free.

## Discussion

This case report underlines the atypical clinical experience of a good outcome in advanced malignant disease. Widespread liver involvement is a major source of morbidity and eventually leads to death in the vast majority of such individuals with poor chances of a radical surgical management, the only available treatment associated with long-term survival. The standard of care for metastatic unresectable colorectal cancer is represented by systemic chemotherapy that can be administered to prolong survival and is considered palliative. Nevertheless, reports of successful resections in these patients following systemic chemotherapy could shift it from being a palliative to a curative treatment. The introduction of monoclonal antibody in a clinical phase II study setting could encourage this intention.

In our patient, the primary treatment was administered for only a short period, much shorter than anticipated and the drug dosages were also decreased in order to reduce the possibility of toxicity events; consequently, discontinuation of therapy was anticipated. Nevertheless, the patient achieved a CR that was later documented to be a pCR with a disease-free survival longer than 36 months and which is still persistent.

A recent study showed that the rates of radical surgery are better in the group of patients receiving bevacizumab together with oxaliplatin-based regimen chemotherapy versus oxaliplatin-chemotherapy alone administered with neoadjuvant purpose [11]. It is notable that this result, achieved despite the short duration of primary treatment, confirms other similar experiences indicating that two to four cycles of the combination of fluoropyrimidine plus oxaliplatin and bevacizumab are not less effective than longer treatment based on cytotoxic therapy without bevacizumab, with regard to pathologic response [12].

It should be noted that primary metastatic cancer patients usually have unfavorable prognosis and palliative chemotherapy has the objective to prolong overall survival. However, pCR was achieved with crucial improvement in the prognosis for the patient.

In clinical practice, an important open debate is the histological confirmation of complete CR. A complete disappearance of metastases on radiological imaging does not always mean a complete disappearance of tumor tissue on pathological examination. The eventuality of pCR should be taken into consideration in all patients with complete CR in order to avoid additional chemotherapy; on the other side, still persistent tumour can undergo to radical surgery in resectable patients. In addition to this, pathologic analysis can represent a possible evaluation of tumour response after prior treatments or hepatic injury of the nontumours liver to cytotoxic therapy.

In our patient, a complete correspondence of radiological imaging was observed. Contrast enhanced CT scan and PET scan did not reveal any liver lesions establishing the reliable sensitivity of different modalities in the evaluation of colon liver lesions after chemotherapy. In our patient, both CT and PET imaging have demonstrated adequate sensitivity to predict pathological response. These congruences, described first between different imaging techniques and secondarily between radiological assessments and the pathologist's report, are not always present and more accurate imaging reflecting the metabolic activity of tumor cells can become necessary.

## Conclusion

The addition of bevacizumab to primary chemotherapy could increase the rate of pCR in liver metastatic CRC patients and may help to improve survival rates in patients with initially unresectable liver disease. Excellent responses to bevacizumab-containing regimes do occur and the referral to surgical oncology is a crucial step for documentation of pCR.

## Competing interests

The authors declare that they have no competing interests.

## Authors' contributions

NM was responsible of the clinical management of the patient, acquisition of data, drafting the manuscript, searching for radiologic imaging; GP was responsible of the scientific revision, discussion and editing of the manuscript; RD, FB, SZ were involved in clinical management of the patient, GL was supervisor of clinical management of the patient and interpretation of data; PFC was principal investigator of phase II clinical trial. All authors read and approved the final manuscript.

## Consent

Written informed consent was obtained from the patient for publication of this case report and accompanying images. A copy of the written consent is available for review by the Editor-in-Chief of this journal

## References

[B1] Pawlik TM, Scoggins CR, Zorzi D, Abdalla EK, Andres A, Eng C, Curley SA, Loyer EM, Muratore A, Mentha G, Capussotti L, Vauthey JN (2005). Effect of surgical margin status on survival and site of recurrence after hepatic resection for colorectal metastases. Ann Surg.

[B2] Adam R, Avisar E, Ariche A, Giachetti S, Azoulay D, Castaing D, Kunstlinger F, Levi F, Bismuth F (2001). Five-year survival following hepatic resection after neoadjuvant therapy for unresectable colorectal liver metastases. Ann Surg Oncol.

[B3] Scheele J, Stang R, Altendorf-Hofmann A, Paul M (1995). Resection of colorectal liver metastases. World J Surg.

[B4] Parikh AA, Gentner B, Wu TT, Curley SA, Ellis LM, Vauthey JN (2003). Perioperative complications in patients undergoing major liver resection with or without neoadjuvant chemotherapy. J Gastrointest Surg.

[B5] Adam R, Pascal G, Castaing D, Azoulay D, Delvart V, Paule B, Levi F, Bismuth H (2004). Tumor progression while on chemotherapy: a contraindication to liver resection for multiple colorectal metastases?. Ann Surg.

[B6] Kuerer HM, Newman LA, Smith TL, Ames FC, Hunt KK, Dhingra K, Theriault RL, Singh G, Binkley SM, Sneige N, Buchholz TA, Ross MI, McNeese MD, Buzdar AU, Hortobagyi GN, Singletary SE (1999). Clinical course of breast cancer patients with complete pathologic primary tumor and axillary lymph node response to doxorubicin-based neoadjuvant chemotherapy. J Clin Oncol.

[B7] Losi L, Luppi G, Gavioli M, Iachetta F, Bertolini F, D'Amico R, Jovic G, Bertoni F, Falchi AM, Conte PF (2006). Prognostic value of Dworak grade regression (GR) in patients with rectal cancer treated with preoperative radiochemotherapy. Int J Colorectal Dis.

[B8] Blazer DG, Kishi Y, Maru DM, Kopetz S, Chun YS, Overman MJ, Fogelman D, Eng C, Chang DZ, Wang H, Zorzi D, Ribero D, Ellis LM, Glover KY, Wolff RA, Curley SA, Abdalla EK, Vauthey JN (2008). Pathologic response to preoperative chemotherapy: a new outcome end point after resection of hepatic colorectal metastases. J Clin Oncol.

[B9] Adam R, Wicherts DA, de Haas RJ, Aloia T, Lévi F, Paule B, Guettier C, Kunstlinger F, Delvart V, Azoulay D, Castaing D (2008). Complete pathologic response after preoperative chemotherapy for colorectal liver metastases: Myth or reality?. J Clin Oncol.

[B10] Benoist S, Brouquet A, Penna C, Julié C, El Hajjam M, Chagnon S, Mitry E, Rougier P, Nordlinger B (2006). Complete response of colorectal liver metastases after chemotherapy: does it mean cure?. J Clin Oncol.

[B11] Cassidy J (2008). Surgery with curative intent in patients (pts) treated with first line chemotherapy (CT) + bevacizumab (BEV) for metastatic colorectal cancer (mCRC): first BEAT and NO16966. (Abstract). Proc ASCO.

[B12] Ribero D, Wang H, Donadon M, Zorzi D, Thomas MB, Eng C, Chang DZ, Curley SA, Abdalla EK, Ellis LM, Vauthey JN (2007). Bevacizumab improves pathologic response and protects against hepatic injury in patients treated with oxaliplatin-based chemotherapy for colorectal cancer liver metastases. Cancer.

